# What drives TFP long-run dynamics in five large European economies?

**DOI:** 10.1007/s40888-021-00215-x

**Published:** 2021-01-22

**Authors:** Alessandro Bellocchi, Edgar J. Sanchez Carrera, Giuseppe Travaglini

**Affiliations:** DESP, Dipartimento di Economia, Società e Politica, Università degli studi di Urbino Carlo Bo, 61029 Urbino, Italy

**Keywords:** Cointegration, TFP, Capital misallocation, Labor misallocation, Scale effects, C22, D21, D24

## Abstract

The aim of this paper is to study the long-run cointegrating relationship of *TFP* in a panel of five large European economies, namely France, Germany, Italy, Spain, and UK. We test whether *TFP* is determined by the so-called “capital misallocation effects, scale effects, and labor market effects”. By considering aggregate data, over the period 1983–2017, we employ dynamic panel cointegration techniques to identify the long-run component of *TFP*. We get two main results. First, the interest rate, the real compensation and the real exchange rate have a positive impact on TFP. Then, the incidence of temporary employment (a proxy of labor market flexibility) has a negative effect on *TFP*. Moreover, for robustness, we run a panel VECM to check for causalities among the variables. Notably, this further excercise confirms the existence of a strong and positive long-run relationship between *TFP* and prices. We conclude that coordinated policies on the issue of interest rate, exchange rate, labour cost and regulation, may allow to reassemble the productivity slowdown puzzle and strengthen the European economic structure.

## Introduction

Total factor productivity (TFP) is the exogenous residual that results from the decomposition of GDP growth (Solow 1957). TFP is generally interpreted as a proxy for technological advancement and productivity, as it also captures the efficiency with which labor and capital are used in production to generate products (Romer 1990). The seminal articles by Prescott (1998) and Edmund (2001) highlight the role of TFP in explaining the income dynamics of countries, as well as differences in international trade, social capital and R&D. Prescott (1998) pointed out, the standard theory of economic growth first needs to analyze the determinants of TFP to also become a theory of international income differences. However, despite its great relevance from a theoretical point of view, there is still no widely shared theory about TFP.

The main motivation of the paper is to deepen our understanding of the factors driving TFP in the long run. We focus on a panel of five major European economies, i.e. France, Germany, Italy, Spain, and UK. We attempt to explain their TFP patterns over the period 1983–2017. By establishing a link among capital misallocation, scale effects and labor misallocation, we show that real prices and labor regulation may have permanent, and sometime adverse effects, on the evolution of TFP in the long run. This is the main contribution of the paper since traditionally the research efforts on TFP have been directed toward technological factors thus leaving out the role of prices. We get two main results. First, the interest rate, the elasticity of saving, the real compensation and the real exchange rate have a positive impact on TFP. Second, the incidence of temporary employment (a proxy of labor market flexibility) has a negative effect on TFP in the long run. Finally, for robustness, we run a panel VECM to check for causalities among variables. Notably, from our empirical analysis emerges a strong and positive long-run relationship between TFP and real prices.

In addition, we derive some intriguing policy implications for the main European economies. Eurozone has shown, over the last 10 years, a marked asymmetry in the pattern and size of economic growth, which appear to be unsustainable in the long run. The identification of common policies for exchange rate, labour cost, regulation, monetary policy, and their possible diversity among countries, can instead allow to reassemble the European puzzle. In fact, equipping any single countries, like those studied in this paper, and their overall set, with coordinated policies— differentiated or shared—is crucial to strengthen the European economic structure, also in the light of the current COVID-19 health emergency.

The paper is organized as follows. In the next section we present a comprehensive review of the literature. Section 2 introduces our database and some stylized facts. In Sects. 3 and 4 we run the empirical model and focus on the main results and policy implications. Finally Sect. 5 concludes with some policy implications.

## Literature

Azariadis and Kaas (2016) developed a theoretical model with AK technology concluding that TFP is of cardinal importance to explain both long-run growth and the business cycle. Particularly, they show how credit market frictions limit capital mobility and slow down the movement of resources from temporarily less to temporarily more productive sectors, and more generally, from temporarily low to temporarily high valuations. Accordingly, Kaas (2016) developed a DGE model exploring the role of public debt on TFP. He shows that a stable equilibrium is one in which a reduction of the primary deficit triggers an expansion of credit and capital, however leading to a deterioration of TFP. Indeed, low-productivity firms may remain active at the lower interest rate, fueling a capital misallocation process which negatively affects the technological process. Choi and Pyun (2017) using firm-level data show that, while an immediate depreciation of the currency leads to an increased productivity through price competitiveness and the expansion of the production scale, persistent and long-term depreciation cancels out productivity gains by slowing down the innovation effort. Bergeaud et al. (2019) argue that the long-run causality from productivity to the real interest rate is only part of the picture. Indeed, since the real interest rate is a determinant of the minimum expected return from investments, the decline in long-run real interest rates may have led to a slowdown in productivity by allowing a growing number of unproductive firms to survive in the market. Finally, Vergeer and Kleinknecht (2010) using panel data analysis show that wage-cost saving flexibilization of labor markets may have a negative impact on labor productivity growth. Dynamics corcening relative prices can ultimately result in a misallocation of capital with a negative impact on TFP. However, empirical studies are still far from reaching a convincing explanation. Recent literature stressed also the relationship between TFP and labor market regulation. Storm and Naastepad (2009) found evidence for a cross-section of twenty OECD countries that a relatively regulated industrial relations system promotes long-run productivity growth.

Productivity is a key factor in raising a country’s standard of living. At least since the early 90s many European economies, especially those of Southern Europe, experienced important structural changes. The slowdown in the growth rate of GDP, the deterioration of labour productivity, TFP and investments are all common features. This negative performance—both from an historical and international perspective—took place in conjunction with a more generalized slowdown in global labour productivity growth. Given this deceleration, a debate has arisen to identify the underlying sources. Some economist argue that the slowdown reflects predominantly cyclical factors, while others point to longer-term structural factors, such as changes in the sectoral composition of the economy, deceleration in the rate of technological progress, and misallocation of factors of production. The picture becomes even more difficult to interpret if we consider the great heterogeneity in productivity levels, the degree of diffusion of new technologies and the quality of human capital in the advanced countries affected by this negative trend.

A first strand of the literature on TFP analyses the contribution of innovative efforts based on research and development to guide the economic system towards its long-term growth path. In addition, productivity improvements could be generated by human capital, whose role in fostering economic growth has been analyzed within the framework of endogenous growth theory. For instance, Lucas (1988) shows that productivity is strongly affected by human capital. This explanation requires attention since many sources of human capital are already incorporated into the inputs which determines TFP, including formal schooling, age, gender, and occupation (Hsieh and Klenow 2009; Restuccia and Rogerson 2017). Actually, a great number of studies stressed the indirect links between human capital and TFP: thanks to specific skills and creative abilities, human capital could facilitate the generation of innovative activities and, consequently, a efficient growth of production (Männasoo et al. 2018).

On the other hand, Reis (2013) found that productivity growth slowed sharply in Portugal and several other economies of southern Europe after they joined the Euro. In his model with credit frictions, financial integration led to a collapse in productivity due to the expansion of relatively unproductive companies in the non-tradable sectors vs the more productive firms in the tradable ones. More recently, Anzoategui et al. (2019) argues that the decline in TFP growth during and after the economic crises of 2008–2010 was a consequence of firms’ responses to the adverse economic cycle. In particular, firms reduced their investments in innovation and technologies, thus speeding up the initial monetary shock. The slowdown in productivity can similarly be rationalized by the sharp decline in R&D activity during the 2001 recession (Aghion et al. 2004; Disney et al. 2003; Foster et al. 2008). Syverson (2011) studies the large differences in productivity within an industry. He conceives productivity as a productive input that differs in quantity or quality across firms, and provides a list of potential determinantes, such as differences in management practice, higher quality labor and capital, differential investment in ICT and R&D, learning by doing, the firm structure, productivity spillovers, regulatory behavior, and differences in the competitive regimes. Thus, TFP growth depends not only on businesses-ability to innovate, but also on the environment that fosters competition, reduces administrative burdens, provides modern and efficient infrastructure, and allows easy access to finance (Bellocchi et al. 2020; Calcagnini et al. 2019). Indeed, real, financial and institutional factors have all been analyzed both from a theoretical and empirical point of view. However, as you may have noticed, research on these issues is rather fragmented and, in only a few cases, analyses the action of these different factors within a unified framework.

A positive relationship between productivity growth and real interest rates is justified on the theoretical ground by growth models *a là* Ramsey (Romer 2012). Productivity is in fact one of the main factors that canto have a significant impact on the return on capital and ultimately on interest rates. However, more recently it has been pointed out that low real interest rates and abundance of credit may lead to poor allocation and weak productivity growth (Bergeaud et al. 2019). In this case, the decline in TFP growth can reflect a misallocation of resources either within and between sectors, which increasingly accounted for the variations of aggregate employment, investments and technology. Cette et al. (2016) confirm this hypothesis and show that productivity growth in Europe was slowing down until 2008. Importantly, they argue that this slowdown was strictly related to monetary policies. The reduction of the real interest rate as a consequence of the euro convergence, led to an unfavorable reallocation of resources which allowed the less productive firms to remain in the market, slowing down the advancement of productivity and TFP. Similarly, Gopinath et al. (2017) show how the decrease in the real interest rates led, in recent years, to a significant decline in sectorial TFP of European economies. Precisely, in response to lower interest rates, capital was misallocated toward firms with lower productive performance, but with higher market value. Another channel working in this direction is the one identified by Liu et al. (2019), according to which a decline in the long-term interest rate can trigger a stronger investment response from market leaders than from market followers, thus leading to greater market concentration, increased profits, and lower aggregate TFP growth.

The literature has also debated whether changes in the real exchange rate and thus competitiveness in international markets affect a country’s total factor productivity. Porter (1990) was one of the first to argue that can be counterproductive for countries to rely on external devaluation as a mean to increase the competitiveness of national firms in international markets. This is because such intervention would discourage firms from seeking a more sustainable competitive advantage in the long run. However, Tomlin and Fung (2010) argue that with persistent exchange rate appreciations, the scale effect on productivity determined by the reduction in the scale of production eventually prevails over the increase in productivity determined by the increased competitiveness. Accordingly, Bagnai and Mongeau-Ospina (2017) sustain that monetary unification—by fixing the nominal exchange rates—contributed to widening divergences in productivity developments in the Euro area. As far as we know, few other studies have considered exchange rates as a determinant of long run TFP in the EU, where, on the other hand, real exchange rate misalignments exist and are even more persistent than in the rest of the world (Fidora et al. 2020).

Although there is a thriving literature on employment protection legislation (EPL) and how it affects the labor market, predictions on its impact on aggregate productivity are ambiguous. The current debate on labour market regulation identifies two main opposite effects. On the one hand, labour regulation increases labour and capital adjustment costs of firms, thus depressing innovation. For instance Conti and Sulis (2016) and Bjuggren (2018) both argue that more flexible labor and product markets are fundamental for a faster reorganization of production resources, thus allowing countries to move towards the production frontier with greater speed. On the other hand, stricter labor regulation can stimulate companies to innovate and invest in R&D and human capital in order to recover productivity and profits in the long run (Calcagnini et al. 2019). Similarly, Riley and Bondibene (2017) found evidence that UK firms responded to increases in labour costs—following the introduction of national minimum wage—by raising labour productivity and without reducing their workforce or substituting capital for labour. Further, the reduced risk of dismissal and the extension of employment contracts may induce firms to encourage the acquisition of more specific skills for their workers with an increase in human capital and eventually productivity (i.e. welfare-improving channel) (Belot et al. 2007).

### A taxonomy

Previous studies highlight that misallocation of productive factors can be induced by changes in prices, and among these, by a low real interest rate. But, monetary policy also affects the nominal (and real) exchange rate. In addition, changes in labor market regulation, and wage policy, can determine changes in labor cost, affecting firms investment decision and their attitude towards innovation (Bellocchi et al. 2020). Therefore, to empirically investigate the TFP determinantes in the long-run, we focus on a large set of variables, usually neglegted in the standard growth models, enhancing the role of "prices".

Specifically, we rely on the following taxonomy:

- *Capital misallocation* It states that persistently low interest rates may undermine TFP growth. In fact, a drop in the real interest rate increases the ability of low competitive firms to operate in the market, reducing their innovation activities, making a low profit and eventually resulting in a poor productivity level.
- *Scale effects* It states that the effect of changes in real exchange rate on TFP, goes through the presence of scale economies. In fact, an overvalued currency may reduce the scale of production, and hence the labor productivity (Verdoorn 1949; Kaldor 1966). We call this relationship "demand-side" view (Travaglini and Bellocchi 2018). Opposite to this interpretation, the "supply-side" view stresses the positive and long-lasting consequences of a real exchange rate appreciation on productivity and TFP. As a consequence, a “hard” real exchange rate may contribute to increase productivity and competitiveness in the long run by forcing innovation and technology progress in tradeable sectors (Porter 1990).
- *Labor misallocation* It focuses on the role of labor market reforms on TFP and productivity. Labor market (de)regulations may have a range of implications for productivity (Saltari and Travaglini 2009). On the one hand, Labor regulations have a negative impact on TFP growth in those industries that rely more on layoffs to adjust the labor force. On the other hand, a less flexible labor regulation and a higher real wage can stimulate the firms to invest and innovate to recover profits, positively affecting TFP and productivity in the long run (Acemoglu 1998; Blanchard and Wolfers 2000; Griffith and Macartney 2014; Pessoa and Van Reenen 2014).

Using country-level panel data, we address these issues for four economies of the EU, namely France, Germany, Italy, Spain, and the UK, over the period 1983–2017. Our aim is to deepen the understanding of the sources of TFP in Europe. Mainly, we focus on the long-run relationship between TFP and its determinants: the real interest rate, the real exchange rate, the real wage and the labor regulations. This is done by adopting fully modified OLS (FMOLS), and dynamic OLS (DOLS) estimators in cointegrated regression models. Finally, for robustness, we run a panel VECM to check for causalities among variables. Notably, from our outcomes emerges a strong and positive long-run relationship between "prices" and *TFP*.

## Database and stylized facts

As mentioned, our dataset consists of a balanced panel for four major economies of the EU and the UK, over the period 1983–2017. The frequency of observations is annual. All the nominal variables were converted into real terms by employing the deflator of GDP. The dataset is based on the most extensive comparable data source at country level, i.e. the European Commission AMECO database. It provides information on:^1^

1. Total Factor Productiity. *TFP* is computed as an index with 2010 = 100. As usual, TFP includes the impact of any input which shifts the production function in the long run. It represents a good proxy for scientific and technological progress.
2. Real Long-Term Interest Rate. *IR* is the interest rates for long-term government bonds denominated in Euro. IR represents the price of intertemporal allocation of goods and thereby determines saving, investment and, ultimately, affects the TFP. In our empirical model is employed to quantify the capital missallocation effect on TFP.
3. Real Effective Exchange Rate. *ER* is computed as a weighted average of the bilateral exchange rates of the euro against currencies of a selection of trading partners. This means that countries with larger trade relationships have higher weights, while countries with smaller trade relationships have lower weights in the basket of currencies. ER provides a measure of the international price competitiveness, which depend not only on exchange rate movements but also on the relative prices of goods and services.
4. Real compensation. *RC* is defined as the total remuneration payd by an employer to an employee in exchange for the work performed. The AMECO version refers to a domestic concept and hence consists of wages and salaries and employers’ social contributions for residents as well as non-residents working for resident producer units.
5. Labor market regulation. We proxy this variable by employing the Incidence of Temporary Employment, *ITE*, on standardized age group 15–24, from the OECD statistics. ITE gives a measure of the effects of changes in labor regulation on TFP. We assume that ITE is positively correlated with labour market flexibility, and hence in a more (less) flexible labor market the share of temporary employment is higher (lower).
6. Gross national saving. *S* is computed by AMECO as a deduction of final consumption expenditure from the gross national disposable income and consists of personal savings, plus business savings, plus public savings, but excludes foreign savings. We use *S* as a control variable in addition to the indicators, to check the robustness of the relationships in our regressions.

All the variables are expressed in natural logarithms, with exception of interest rate and the incidence of temporary employment (which are already in percentage units). The log transformation is important in order to decrease the variance among variable and obtain efficient estimators. Descriptive statistics are shown in Table 1.

**Table 1:** Descriptive statistics

	TFP	ER	IR	ITE	RC	S
Mean	4.5475	4.5972	3.2403	39.1399	4.4717	5.3760
Median	4.5848	4.6008	3.3837	40.9100	4.5170	5.4579
Maximum	4.6564	4.8971	8.5388	76.4900	4.6858	6.7701
Minimum	4.2577	4.3280	$-$5.2258	9.0100	3.9809	3.5357
Std. Dev.	0.0888	0.1093	2.1619	20.8743	0.1454	0.6582
Skewness	$-$1.0655	0.0474	$-$0.5319	$-$0.0544	$-$1.3039	$-$0.4346
Kurtosis	3.3540	3.0077	3.9593	1.6244	4.4892	3.1317
Jarque-Bera	35.9748	0.0698	15.8192	13.0891	69.5191	5.9588
Probability	0.0000	0.9656	0.0003	0.0014	0.0000	0.0508
Sum	841.294	850.4835	599.4651	6458.09	827.27	994.5664
Sum Sq. Dev.	1.4524	2.2003	860.0275	71461.4	3.8904	79.7342
Observations	170	170	170	170	170	170

Source: Summary statistics of panel data (1983–2017). Own elaboration on AMADEUS and OECD

**Fig. 1:**
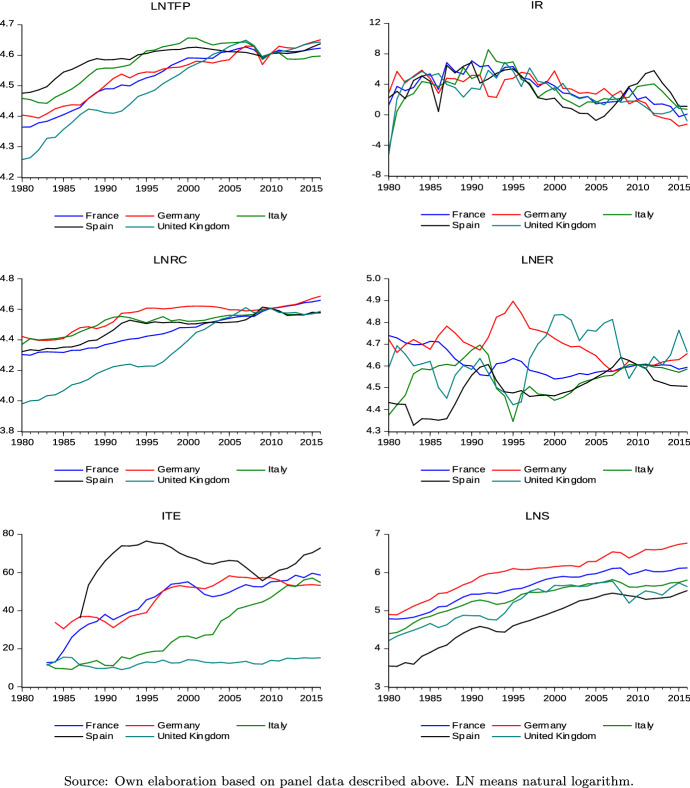
France, Germany, Italy, Spain, UK: trajectories and trend (1983–2017). Source: own elaboration based on panel data described above. LN means natural logarithm

Figure  1 shows the pattern of the variables. Starting from TFP we observe a growing trend since 1980 common to all the economies with a deceleration over the last decades. In European countries—but more generally in all advanced economies—TFP records a considerable slowdown after the early 2000s, and a significant fall in 2008 with a weak recovery only in the more recent years until 2017. Notably, a brilliant performance is observed only in Germany. The same figure also illustrates a significant decline in real long-term interest rates starting from the late 1990s. This trend in Europe echoes a more general and constant downward trend around the world over the last 30 years. More specifically, the long term interest rate for safe and liquid assets raised in UK and Italy during the early 80s, then stabilized in the 2000, falling steadily until the most recent years. They reached historical low levels in the aftermath of the global crisis and have not recovered since then—pushed down by the a weak economic growth. In 2017 the real long-term interest rate is negative in three out of the five countries of the sample and varies from 1.70 in Italy to $-1.10$ in Germany. It is interesting to note how the standard deviation decreased until 2008, exploded during the crisis, to converge towards its initial level under the influence of the ECB monetary policy. We observe a positive but weak growing trend for real compensation. Wages grew in every economy, albeit at different speeds between 1980 and 2000. The average growth rate over the period was 0.84% with a range from the 0.60 in Italy to the 0.97 in France. The analysis shows that nominal wage growth has closely tracked inflation and productivity since the mid-1990s, but the link between wages and prices has weakened after the crisis due to weak inflationary pressures. Notably, a slowdown characterizes Germany which—together with Italy—is a country characterized by relatively low or moderate wage growth.These countries, together with the UK, recorded significantly lower nominal wage growth in the post-crisis period than one would be expect from their economic fundamentals. Stagnation of real compensation was associated to high unemployment, rising pressure on the welfare state and wage moderation policies (European Commission 2017). Finally, a common feature of these European countries is their saving capacity. From the inspection of Fig.  1 emerges an increasing trend for gross national savings. However, there are three main changes in the slope of the curve that are worth dwelling on. A first phase (until 1995) of constant growth at an average rate of 8.5% (5% in Germany and 11% in Italy and Spain). A subsequent slowdown to an average of 5.5%, between 1995 and 2007, and finally a stagnation in the more recent yars common to all countries, but Germany. In most of the cases national savings were lower than the total investment, resulting in increasing foreign debt and a negative current account. The fall in household savings has been paricularly marked in Italy. This latter country—which was a high-savings one until the mid-1990s—has seen its household saving rate fall since then (Campiglio 2013).

**Fig. 2:**
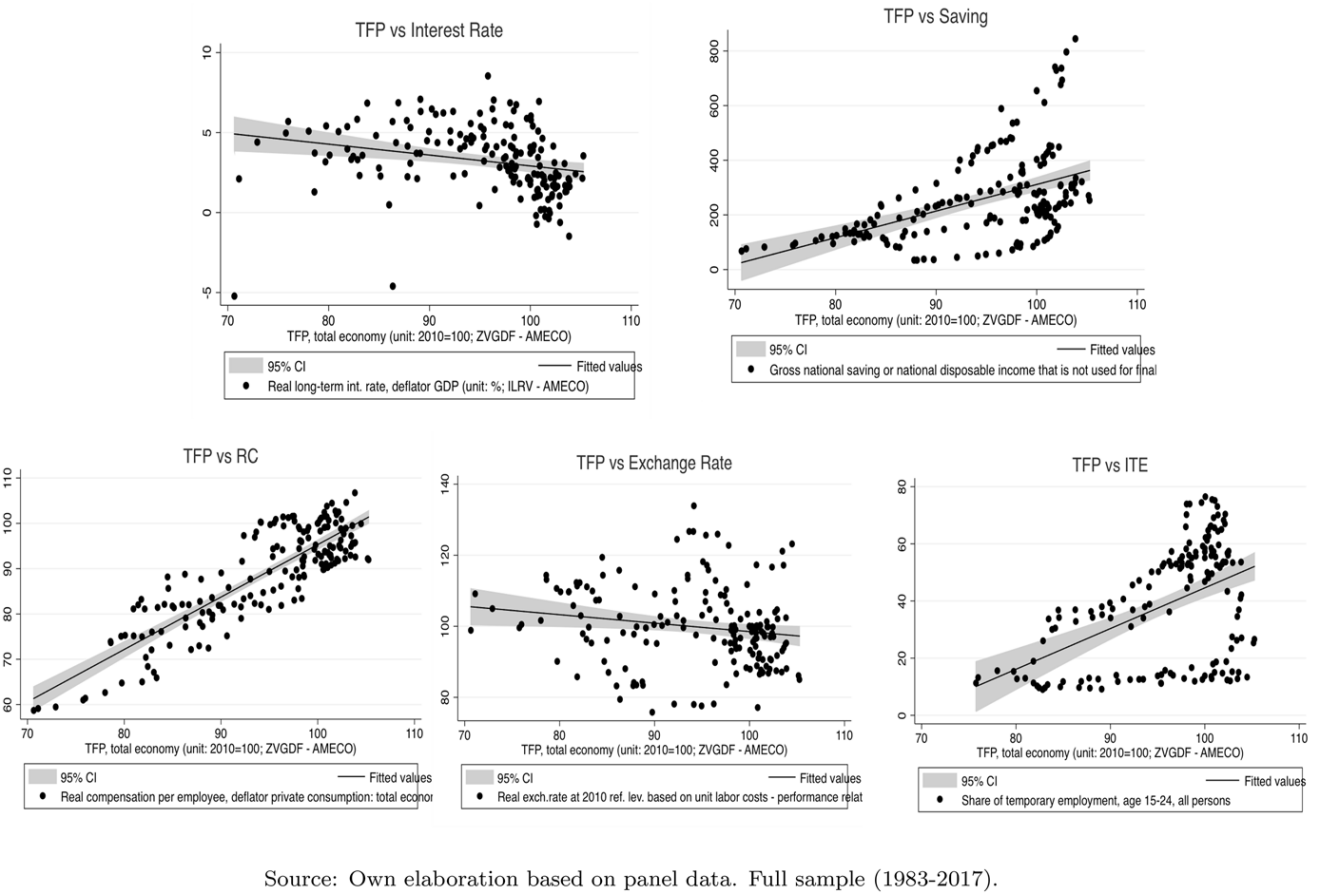
Pairwise scatter graphs: TFP vs independent variables (IR, S, RC, ER, ITE). Source: own elaboration based on panel data. Full sample (1983–2017)

Figure  2 plots the scatter between the TFP and the other variables: IR, ER, RC, ITE and S. These panels provide information on the positive and negative relationship between the TFP and its potential explanatory factors. As we can observe, in some cases the linear regression lines do not capture all the information spread in the clouds. However, the relationships between TFP-IR, TFP-S and TFP-RC are statistically significant.

The existence of a strong and significant correlation between these variable and technological progress may potentially account for variations in TFP both in cross-country and time-series perspective. Therefore, starting from these stylized facts, and some recent theoretical and empirical contributions (Bellocchi et al. 2020), we estimate two different specifications where interest rate and saving are employed alternatively to by-pass their positive correlation and the potential distorting effect of it on the dynamics of TFP. We specify the TFP relationship as follows:

1 $$\begin{aligned} TFP_{it}=f(IR_{it},ER_{it},RC_{it},ITE_{it}) \end{aligned}$$

where $\epsilon _{it}$ is the error term with all unobserved factors, *it* denotes the observation on the $i-th$ cross-section unit at time *t*, for $t=1,2,\ldots ,T$ and $i=1,2,\ldots N$. Then, we further expand equation (1) to obtain a model that will provide the basis for our empirical analyisis and will be estimated using two dynamic panel data techniques, namely Fully Modified OLS (FMOLS) and Dynamic OLS (DOLS).

2 $$\begin{aligned} lnTFP_{it}=\beta _{0i}+\beta _{1i}IR_{it}+\beta _{2i}lnER_{it} +\beta _{3i}lnRC_{it}+\beta _{4i}ITE_{it}+\epsilon _{it} \end{aligned}$$

In the next section, we use this specification to show that the correlations found in stylised facts are robust to a number of econometric issues and the inclusion of control variables.

## Panel cointegration analysis

Before carrying out the cointegration tests, it is important to verify the order of integration of the variables. Actually, since many of our macroeconomic variables are trended, time series estimation techniques can potentially provide spurious correlation in presence of non-stationarity (Phillips 1986). Therefore, our cointegration analysis is conducted in four steps. (1) First, panel unit root tests are applied to examine whether the variables included in the model are stationary. (2) Next, panel cointegration tests are run to understand if an equilibrium relationship exists between single non-stationary variables. (3) Then, when a potential cointegration relationship is determined, this latter is eventually estimated by means of panel FMOLS and DOLS techniques. (4) Finally, we apply the panel VECM to investigate the direction of the causal relationship among cointegrated variables.

### Panel unit root tests

Several unit root tests have been proposed by the literature. Thus, in order to verify the presence of unit roots and avoid spurious regression, we perform a battery of them. Among the many, we rely on those developed by Levin et al. (2002), Im et al. (2003) and both ADF and PP—Fisher type unit root tests proposed by Maddala and Wu (1999).^2^ In particular, we employ LLC since it can be used as pooled panel unit root test, IPS to allow for heterogeneity, and MW for the non-parametric approach. First generation panel unit-root tests includes both individual unit root tests and common unit root tests. The main difference between the two is the assumption of common or different AR coefficients in each series. Hence, alternative hypothesis of common unit root tests are homogenous across all units whereas individual unit root tests permit some group to be stationary and some are nonstationary in alternative hypothesis. The IPS and MW tests are individual unit root tests while LLC is a panel unit root test. As it is well known, panel based unit root test are preferred to individual ones. However the IPS combines information from either the cross-sectional and time series dimension, such that fewer observations are required for the test to have power (Campbell and Perron 1991).

Table 2 displays results of the panel unit root tests for each variable in our panel of countries. The tests were performed both on the variables in level as well as in their first differences. In these tests, the null hypothesis is that the variable contains a unit root (i.e., it is not stationary). Further, the tests have been carried out with two different regression specifications, one with constant and the other with a constant and a linear trend.

**Table 2:** Panel unit root tests

	TFP	ER	IR	ITE	RC	S
	P-value	P-value	P-value	P-value	P-value	P-value
Assumption: individual effects (constant term), individual linear trends						
Test for unit root of variable in level						
Null: assumes common unit root process						
Levin, Lin & Chu t	0.0619	0.3500	0.6228	0.8237	0.6052	0.265
Breitung t-stat	0.7472	0.0590	0.5669	0.5670	0.4390	0.82
Null: assumes individual unit root process						
Im, Pesaran and Shin W-stat	0.8356	0.1519	0.0966	0.6659	0.7489	0.925
ADF—Fisher Chi-square	0.7485	0.2602	0.1163	0.6924	0.8297	0.9596
PP—Fisher Chi-square	0.9817	0.7564	0.0000*	0.0731	0.3058	0.9906
Test for unit root of variable in 1st difference						
Null: assumes common unit root process						
Levin, Lin & Chu t	0.0000*	0.0094*	0.0000*	0.0805*	0.0000*	0.0000*
Breitung t-stat	0.0000*	0.0000*	0.0000*	0.0004*	0.0000*	0.0000*
Null: assumes individual unit root process						
Im, Pesaran and Shin W-stat	0.0000*	0.0000*	0.0000*	0.0000*	0.0000*	0.0000*
ADF—Fisher Chi-square	0.0000*	0.0001*	0.0000*	0.0000*	0.0001*	0.0000*
PP—Fisher Chi-square	0.0000*	0.0000*	0.0000*	0.0000*	0.0000*	0.0000*

* Null Hypothesis Rejection

P-values for the log-levels of TFP, IR, ER, ITE, RC and S are insignificant, implying that each of the five variables is non-stationary—the null hypothesis of a unit root cannot be rejected at the standard level of 5%. However, when we apply the unit root tests to variables in first differences the null hypothesis of a unit root (either with an intercept or intercept/trend) is rejected at the 1% significance level. Therefore, according to these outcomes, we can conclude the six variables of our model contain a panel unit root. In other words, time series are integrated processes of order one, *I*(1), and a possible long run cointegrating relationships may exist.

### Panel cointegration

A cointegration analysis is performed in order to test the possibility of a long run convergence of our variables. Panel cointegration tests combine information on similar long-run relationships while allowing for heterogeneous short-run fluctuations and fixed effects among panel members. Considering such heterogeneity offers important advantages, as it would be restrictive to assume that the vectors of cointegration are similar in all panel members (Pedroni 1999). For the robustness of our analysis we employ three different types of panel cointegration tests: the first one was introduced by Pedroni (1999, 2004), a second type was proposed by Kao (1999) and is based on Engle and Granger (1987) test, while finally a third one was developed by Fisher and Maddala and Wu (1999), by adjusting the Johansen test to panel data:

3 $$\begin{aligned} \Delta y_{it}= \Pi _iy_{it-1}+\sum ^k_{j=1} \lambda _{ij} \Delta y_{it-1}+ \phi _i z_{it} + \epsilon _{it}, \end{aligned}$$

where $y_{it}$ is a *px*1 vector [TFP, ER, IR, ITE, RC], *p* is the number of variables and $\Pi _i$ represents the long-run $p \times p$ matrix. If $1<rank(\Pi _i)<p$, the matrix can be written as $\alpha _i \beta '_i$, where $\beta '_i$ is a $r \times p$ matrix which rows are the cointegrating vectors, while $\alpha _i$ is a $p \times r$ matrix that gives the amount of each cointegrating vector in the error correction model. Johansen’s test includes two different statistic which are obtained respectively by summing the p-values of the cross sectional trace or maximum eigenvalue cointegration tests. Both forms of the test will determine if cointegration is present. The null hypothesis is always that there are no cointegrating equations.^3^ Note that once it is established that the variables are cointegrated, before the estimation of the long run model to obtain the elasticities it is necessary to determine the number of cointegration relationships (Johansen 2000). Indeed, one advantage of these tests is that they do not specify the cointegration vectors, but simply identify how many stationary combinations can be obtained with the given set of variables. This means that once it is concluded that there are respectively 1, 2 or *n* vectors of cointegration, there is still the problem of identifying them. However, this can be easily solved by calculating Pedroni’s (1999) ADF and PP test statistics within and between dimensions. The results of panel cointegration tests are reported in Tables 3 and 4.

**Table 3:** Panel cointegration Ttests

Model 1: TFP, ER, IR, ITE, RC			Model 2: TFP, ER, ITE, RC, S		
Null Hypothesis: No cointegration			Null Hypothesis: No cointegration		
Kao Residual Cointegration Test: Summay			Kao Residual Cointegration Test: Summay		
ADF (Augmented Dickey-Fuller Test):	t-Statistic	P-value	ADF (Augmented Dickey-Fuller Test):	t-Statistic	P-value
	− 3.085303	0.0010*		− 3.177452	0.0007*
Residual variance: 0.000166			Residual variance: 0.000079		
HAC variance: 0.000199			HAC variance: 0.000077		
Pedroni Residual Cointegration Test: Summary			Pedroni Residual Cointegration Test: Summary		
Alternative hypothesis: common AR coefs. (within-dimension)			Alternative hypothesis: common AR coefs. (within-dimension)		
	Statistic	P-value		Statistic	P-value
Panel PP-Statistic	− 1.312961	0.0946*	Panel PP-Statistic	− 1.47029	0.0707*
Panel ADF-Statistic	− 1.313852	0.0944*	Panel ADF-Statistic	− 2.870269	0.0021*
	Weighted Statistic	P-value		Weighted Statistic	P-value
Panel ADF-Statistic	− 1.631689	0.0514*	Panel ADF-Statistic	− 1.79215	0.0366*
Alternative hypothesis: individual AR coefs. (between-dimension)			Alternative hypothesis: individual AR coefs. (between-dimension)		
	Statistic	P-value		Statistic	P-value
Group ADF-Statistic:	− 1.550644	0.0605*	Group ADF-Statistic:	− 2.054842	0.0199*

* Null Hypothesis Rejection. Lag length selection based on AIC and SC: lag of 1. Newey-West automatic bandwidth selection and Bartlett kernel

**Table 4:** Johansen Fisher Panel Cointegration Test

Series: TFP, ER, IR, ITE, RC					Series: TFP, ER, ITE, RC, S				
Trend assumption: Linear deterministic trend					Trend assumption: Linear deterministic trend				
Lags interval (in first differences): 1 1					Lags interval (in first differences): 1 1				
Unrestricted Cointegration Rank Test (Trace and Maximum Eigenvalue)					Unrestricted Cointegration Rank Test (Trace and Maximum Eigenvalue)				
Hipothesized no. of cointegrations	Fisher Stat.**		Fisher Stat.**		Hipothesized no. of cointegrations	Fisher Stat.**		Fisher Stat.**	
	(From trace test)	P-value	(From max-eigen test)	P-value		(From trace test)	P-value	(From max-eigen test)	P-value
None	71.46	0.0000*	45.82	0.0000*	None	74	0.0000*	41.22	0.0000*
At most 1	34.25	0.0002*	20.81	0.0224*	At most 1	41.92	0.0001*	25.08	0.0052*
At most 2	19.06	0.0395*	14.91	0.1353	At most 2	20.32	0.0568	12.48	0.2542
At most 3	11.1	0.3494	7.999	0.6289	At most 3	18.74	0.3786	6.411	0.7796
At most 4	17.59	0.0623	17.59	0.0623	At most 4	19.28	0.305	11.71	0.305

* Null Hypothesis Rejection

**Probabilities are computed using asymptotic Chi-square distribution

As clearly emerges from the Kao test (ADF statistic) we can significantly reject the null hypothesis of no cointegration among our variables. Similarly, from the Pedroni cointegration test we can reject the null hypothesis of no cointegration at the 0.1% level, regardless linear trends are included. Therefore, it is very likely that there is a long run co-movement between TFP and the dependent variables. Further, Johansen Fisher max-eigen results are such that we cannot reject to have at most 2 (up to 5) cointegrating relations, while from Johansen Fisher trace test test we cannot reject to obtain at most 3 (up to 5) cointegrating relations.

### Panel FMOLS and DOLS estimators

Since unit root and cointegration tests suggest that the variables are non stationary and cointegrated, we attempt to estimate the long-run equilibrium relationship. Indeed, although the cointegration tests allow us to verify the presence of cointegration, they cannot provide an estimate of the underlying long-term relationship. Banerjee et al. (1993) highlight the important connection between a cointegration relationship and the corresponding long-run equilibrium equation. Indeed, the search for a co-integration relationship is the search for a statistical equilibrium between variables that tend to grow over time. The presence of endogeneity in our data and the possible correlation among errors may result in the dependence of OLS estimators. For this reason a standard OLS estimator would be inconsistent and biased in cointegrated panels (Apergis et al. 2007). We consider two approaches to estimating a long-run relationship: Fully Modified Ordinary Least Squares (FMOLS) and a Dynamic Ordinary Least Squares (DOLS) estimation for the between-dimension “group mean”. These estimators allow us for a larger flexibility in the presence of heterogeneity in the examined cointegrated vectors (Pedroni 1999, 2000, 2001, 2004). FMOLS is a nonparametric correction which considers adjustments for autocorrelation by taking into account of the possible correlation between the error term and the first differences of the regressors, as well as the presence of a constant. On the other hand, DOLS is a parametric approximation where the delayed terms in first differences are explicitly estimated. With DOLS, errors are augmented with advanced, delayed and contemporaneous values of the regressors to eliminate the feedback in the cointegrating system (Saikkonen 1992 and Stock and Watson 1993). We estimate the long run relationship using the dynamic ordinary least squares (DOLS) within-dimension (pooled) estimator suggested by Kao and Chiang (2000). We opted for this estimator since it yields unbiased and asymptotically efficient estimates of the long run relationship, even if there are endogenous regressors, thus allowing us to control for the potential endogeneity. Our baseline specification considers the following cointegrated system for a panel of *i = (France, Germany, Italy, Spain, and UK)* countries, over the period $t=1983-2017$.

4 $$\begin{aligned} y_{it}=  \alpha _i+\beta x_{it}+\mu _{it} \end{aligned}$$

5 $$\begin{aligned} x_{it}=  x_{it-1}+\epsilon _{it} \end{aligned}$$

where $y_{it}$ is our dependent variable *TFP*, $\beta$ is a $k\times 1$ vector of the slope parameters, $x_{it}$ is a vector that has as elements the regressors [IR, ER, RC, ITE, S], and that the remaining idiosyncratic error $\mu _{it}$ is independent across *i* but possibly dependent across *t*, while the vector error process $\xi =(\mu _{it}, \epsilon _{it})'$ is stationary with asymptotic covariance matrix $\Omega _i$. Thus, the set of variables $x_i$, $y_i$ are said to cointegrate for each member of the panel, with cointegrating vector $\beta$ if $y_{it}$ is integrated of order one. The composite equilibrium error $y_{it}-\beta x_{it}$ is potentially comprised of an individual-specific effect $\alpha _i$. So the term $\alpha _i$ allows the cointegrating relationship to include member specific fixed effects. By examining the limiting distribution of the FMOLS and DOLS estimators, Kao and Chiang (2000) show that they are asymptotically normal. FMOLS estimator is defined as:

6 $$\begin{aligned} \beta _F^{*}=\left[ \sum _{i=1}^N \sum _{t=1}^T (x_{it}-\bar{x}_i)' \right] ^{-1} \left[ \sum _{i=1}^N \left( \sum _{t=1}^T (x_{it}-\bar{x}_i)\bar{y}_{it} + T\Delta _{\epsilon \mu }\right) \right] , \end{aligned}$$

where $\Delta _{\epsilon \mu }$ is the serial correlation term and $\bar{y}_{it}$ is the transformed variable of $y_{it}$ to achieve the endogeneity correction. The serial correlation and the endogeneity can also be corrected by DOLS estimator which includes the past and the future values of the differenced I(1), i.e. the coefficient of a lead or lag of first differenced regressors. DOLS estimator is given by:

7 $$\begin{aligned} \beta _D^{*}=\sum _{i=1}^N \left( \sum _{t=1}^T z_{it} z'_{it} \right) \left( \sum _{t=1}^T z_{it} \bar{y}_{it} \right) , \end{aligned}$$

where $z_{it} =[x_{it}-\bar{x}_i, \Delta x_{i, t-q},\ldots , \Delta x_{i, t+q}]$ is $2(q+1)$ vector of regressors. While FMOLS takes into account for “serial correlation effects” and “endogeneity” in the regressors resulting from the existence of a cointegrating relationship, DOLS deals with the problem of second-order asymptotic bias arising from serial correlation and endogeneity. Both of them are asymptotically equivalent and efficient. Mark and Sul (2003) show that panel DOLS is fully parametric (relevant test statistics have standard asymptotic distributions) and offers a computationally convenient alternative to the panel FMOLS estimator proposed by Pedroni (1997) and Phillips and Moon (1999).^4^ To correct the endogeneity of regressors and serial correlation, Pedroni (2000) proposes an FMOLS group mean estimator that incorporates the semi-parametric correction of Phillips and Hansen (1990).^5^ In a the same spirit of the nonparametric FMOLS, Pedroni (2001) has constructed a group mean panel DOLS estimator between groups that incorporates parametric correction for endogeneity and autocorrelation. We show our results (panel grouped mean) in Tables  5 and  6 below. Coefficients are obtained as cross-country average of the individual cross-country long-run estimation. The period studied is 1983–2017. Due to the strong correlation between the interest rate and savings, we present two models, i.e. (1) model 1 includes the interest rate without the saving variable and (2) model 2 includes saving without the interest rate.

**Table 5:** Panel Fully Modified Least Squares (FMOLS)

Dependent Variable: LNTFP				
Variable	Coefficient	Std. Error	t-Statistic	Prob.
Model 1				
IR	0.007736	0.002099	3.685443	0.0003
ER	0.120105	0.057094	2.39074	0.0370
RC	0.895068	0.061145	14.60718	0.0000
ITE	− 0.000545	0.000885	− 0.615655	0.5390
Model 2				
S	0.046143	0.013076	3.528829	0.0005
ER	0.187940	0.029673	6.333664	0.0000
RC	0.774573	0.043737	17.70974	0.0000
ITE	− 0.001299	0.000611	− 2.126988	0.0350

Panel method: Grouped estimation

Cointegrating equation deterministics: DUMMY0708

**Table 6:** Panel Dynamic Least Squares (DOLS)

Variable	Coefficient	Std. Error	t-Statistic	Prob.
Model 1. Dependent Variable: TFP				
IR	0.006658	0.002077	3.206089	0.0018
ER	0.136176	0.059788	2.277638	0.0247
RC	0.858162	0.062263	13.78282	0.0000
ITE	0.000830	0.001168	0.710590	0.4788
Model 2				
S	0.093683	0.023861	3.926143	0.0002
ER	0.389319	0.064659	6.021111	0.0000
RC	0.491534	0.090188	5.450118	0.0000
ITE	$-0.000353$	0.001419	$-0.248910$	0.8040

Panel method: grouped estimation . Cointegrating equation deterministics: DUMMY0708 . A dummy variables for the Great Recession of 2007-2008 is included

Tables 5 and 6 contain the estimation results of long run relationship between TFP and IR, ER, RC, ITE. They can be summarized as follows:

- The FMOLS estimation indicates that real long-term interest rate, *IR*, is highly statistically significant and it has a positive effect on TFP, although the impact is small (0.0077). The DOLS estimation produces a positive statistically significant effect of IR (0.0066) on TFP as well. This is consistent with our hypothesis of capital misallocation, that a drecreasing of real interest rates can negatively affect TFP reducing investments and innovation in the long run.
- The effect of real effective exchange rate, *ER*, is positive and statistically significant by applying either FMOLS or DOLS estimators. The long-run elasticity is positive. A 1% increment in ER increases by roughly 0.20% the long-run level of TFP. This is in line with our "supply side view" of the real exchange rate that the appreciation of the national currency forces companies to compete harder, resulting in a scale expansion of surviving firms which in turn generates an aggregate increase of the TFP.
- Real compensation per employee, *RC*, has a strong positive and significant effect on TFP in both the models. The long-run coefficient is around 0.8, meaning that an increment in one percent of the compensation of employees increases the long-run TFP by around 0.8%. This effect is strictly connected with our hyphotesis of "labor misallocation", where a fall in the real labor cost induce firms to reduce their capital to labor ratio negatively affecting TFP (Kaldor 1957).
- Labor market flexibility, *ITE*, has a low but small negative effect ($-0.0012$) and it is statistically significant at the 5% level applying FMOLS and using gross saving as regressor (Model 2). This negative effect is strictly connected to wage flexibility which rises the chance for less innovative firms to survive in the market by paying lower wages. While in the short run employment will benefit from firm survival, in the long run it will eventually suffer because wage moderation slowdowns the incentive to renew the capital stock. Also, this phenomenon can be traced back to the assumption of "labor misallocation".
- Gross national saving, *S*, is highly statistically significant at 1% level in FMOLS and DOLS estimations, with respectively a coefficient of 0.046 and 0.093—meaning a positive impact on the long-run dynamics of TFP.^6^

### VECM analysis

Cointegration analysis allow us to establish the existence of a causal relationship between the series considered but does not provid any indication about causality.^7^ Therefore, the last step of our analysis consists in running causality tests. For this purpose we employ a panel-based VECM (vector error-correction model) to identify the existence and direction of a long-term equilibrium relationship (Pesaran et al. 1999).^8^ One of the advantages of panel causality is that it allows to capture effects between variables even considering possible feedbacks. Granger Pairwise causality means that a variable *Y* Granger causes another variable *X* if at time *t*, $X_{t+1}$ can be better predicted by employing past values of *Y* rather than not (Granger 1969). The VECM is a VAR with the long-run relationship showing how variables tends to return to their equilibrium after suffering a shock. In order to obtain the optimal VECM, the lag is selected using Information Criterion.^9^ To test for causality among variables, we specify the following Vector Auto Regressive (VAR) model for cross-section of country *i* in period *t*:

8 $$\begin{aligned} X_{i,t}= \left( TFP_{i,t}, IR_{i,t}, S_{i, t}, ITE_{i, t}, RC_{i, t}, ER_{i, t}\right) \end{aligned}$$

Vector (8) can be represented as a vector error-correction in its first-differenced form (see, for instance, Groen and Kleibergen 2003), i.e.:

9 $$\begin{aligned} \Delta X_{t}=\mu +\Pi X_{t-1}+\sum _{i=1}^{i=k-1}\Gamma _{i}\Delta X_{t-i}+\varepsilon _{i} \end{aligned}$$

where $\mu$ is a vector of constant terms and $\Gamma _{i}$ is the short-run dynamics matrix which is assumed to be unrelated between cross-sections. In the short run $X_{t-i}$ does not Granger cause $X_t$ if $\forall i = 1,\ldots , k-1$, $\Gamma _{i}=0$. The matrix $\Pi$ is the lagged error-correction term derived from the long-run cointegrating relationship, and so conveys information about the long-run relationship between the *X* variables. The rank of $\Pi$ is the number of linearly independent and stationary linear combinations of the variables. The presence of long-run causality can be established if $\Pi$, the coefficient of error correction term $X_{t-1}$ equals to zero. With respect to the VECM (9), short-run causality is determined by the statistical significance of the partial Wald $F-statistics$ associated with the corresponding differenced right hand side variables. Long-run causality is revealed by the statistical significance of the respective (lagged) error correction terms using a $t-test$ and is derived from the long-run cointegrating relationship (Holtz-Eakin et al. 1988: Narayan et al. 2008). If the right side of the VECM equations is similar in all equations, the least squares estimator can be employed without any loss of efficiency (Hamilton 1994). In addition, if all data sets are stopped at the first difference, the least squares estimator is also appropriate. Since our study meets both of these conditions, we can therefore proceed to estimate the equations of the VECM model with the least squares estimator. Furthermore, to validate the results, we use the Wooldridge (2002) test for the serial correlation of panel data. The estimation is repeatedly conducted using two Models. F-statisticsfor the test of serial correlation rejects the null hypothesis ofpresence of serial correlation, hence, the VECM model is wells pecified and conclusion can be drawn from the results.

**Table 7:** Panel Granger causality test by VECM

Model 1			Model 2		
	Short-run Chi-square statistics	Long-Run coefficients		Short-run Chi-square statistics	Long-Run coefficients
Source of Causation ($H_0$: independent variable does not Granger cause TFP)					
IR	3.339755	0.09557** (0.04088) [− 2.33773]	S	1.037645	0.730999** (0.34007) [ 2.14956]
ITE	0.676958	0.005025 (− 0.00355) [ 1.41563]	ITE	0.588168	0.013820 (− 0.0077) [-1.79562]
RC	1.605704	0.287417 (0.62638) [ 0.45886]	RC	3.053111	3.011777 (− 1.60776) [ 1.87328]
ER	3.056762	1.323731** (− 0.61345) [− 2.15785]	ER	2.890318	7.735098** (− 1.72294) [− 4.48947]
All	8.783315	ECT: − 0.015931*** (p-value: 0.0000)	All	8.476453	ECT: − 0.003605*** (p-value: 0.0000)
Weak exogeneity test—Chi-square: 33.03985 P-value: 0.000001			Weak exogeneity test—Chi-square: 17.96903 P-value: 0.000446		

Standard errors in ( ) & t-statistics in [ ]. ***, **, * Significance at 1%, 5% and 10%, respectively

Results of the Granger causality tests based on the panel VECM are reported in Table 7 and show that:^10^

- In both the specifications there is no short-run causality (single and whole variables) ranging from independent variables to TFP.
- There is an individual long-run causality (significant at the 5% level) going from *IR*, *ER* and *S* to TFP common to the two specifications.

Since the error correction term (ECT) is negative and statistically significant we can state that a long-run causality in the system exists, running from the variables (IR, S, ER, RC, ITE) to the dependent variable TFP.

Finally, note that the ECT represents the speed of adjustment towards the long-run equilibrium of the TFP. However, the specification of the model affect this speed which is higher in the first model (1.5% a year) and smaller in the second one where the speed of adjustment is around 0.3% a year.

### Policy implications

There is no consensus among economists as to the causes of the productivity slowdown in advanced economies, and specifically in the EU. On the other hand, it has been recognized the importance of TFP evolution on economic growth, but also on business cycle and unemployment. Several studies seem to suggest that the phenomenon of TFP slowdown may be temporary, and that productivity may accelerate again, although it is not yet clear when (Mokyr et al. 2015; Brynjolfsson and McAfee 2014; Branstetter and Sichel 2017; Bergeaud et al. 2017).

In such a scenario, we have studied the long-run dynamics of TFP with the aim of identifying the role of prices in affecting its evolution. This is a new perspective compared to the standard literature. For the major European countries we found that TFP has a positive relationship with the real interest rate, the real exchange rate, the real compensation of labor and the labor market regulation. What are the policy implications of this result? Mainly, it suggests that not only innovation and investments but also real prices affect the decision of firms and should affect policy makers strategy about technology advancements.

This relationship implies that capital misallocation and labor misallocation could negatively affect TFP in the long run by allowing an increasing number of weakly-productive firms to survive. On the other hand, the positive long-run relationship between TFP and real exchange rate pertains to the "supply-side view" according to which a hard currency can induce firms to update technology and knowledge in order to recover competitiveness in the long run.

Therefore, the strategy to relaunch TFP should not only focus on basic and applied research, human capital and innovation, but also on a price policy coherent with economic growth. As shown, low real interest rates, or exchange rate devaluations, lead firms to postpone investments and innovations to the future. Wage moderation can have analogous effects. Similarly, market deregulation can reduce incentives to invest rather than offer new ones. Our results can be interpreted as "warning signals" with respect to economic policies that aim, in the short run, to maximise political consensus, often myopic to long-term perspectives.

This recommendation is all the more important, the more serious the fall in TFP will be. A comprehensive price policy pushing the economic system to increase the capital to labour ratio, and technological content of production, is just as important as direct innovation and its financing. In this view, the EU Recovery Plan will be successful the more it will be supported by a coherent price policy in the near future for all the European countries.

## Concluding remarks

TFP growth has been slowing down in all major European countries, particularly in Italy, at least since mid 1990s. Understanding the sources of this worrying drift, and the cross differences among similar countries, is of crucial interest to any single government and EU policy maker.

We shown how the patterns of TFP in the major economies of the Eurozone—Italy, Germany, France and Spain—and the UK, can be traced back to three main shocks to the: real interest rate, real exchange rate, and cost and regulation of labour. To address this analysis, we employ macro data and study the aggregate effects of capital and labor misallocation on TFP, over time, in these economies. Such a shocks can have either permanent and temporary effects. Further, they can have unexpected and unintended consequences in the long run. We use a simple taxonomy to order the literature on the issue.

Using a panel data analysis and a VECM procedure we get several results. First, we show that misallocation of capital and labour can adversely affect TFP growth in the long run. Precisely, TFP growth shows a positive relationship with price changes in the long run, but it may be biased along the cycle. Second, we found a positive long-run relationship between TFP and real exchange rate. This result strengthens the "supply-side view" of the relationship between productivity and real exchange rate, according to which a hard currency can induce firms to update their technological constraints in order to recover competitiveness and profitability in the long run. Third, deregulation in labour market, together with wage moderation, reducing rule and labour costs, and increasing profits in the short run, discourages firms from investing and innovating at the current time, thus contributing to the deterioration of TFP in the long run.

To summarise, this research offers new and relevant information on the relationship between technological progress and prices, and can be further extended to disentangle, at sectoral and firm level, the role of prices on TFP, investment and innovation. These are all possible objectives to pursue in our next study, both by refining the econometric model and by providing a sector-based economic model.

## Footnotes

1 Following AMECO’s nomenclature and codes, we use the following series: total factor productivity (ZVGDF); real long-term interest rates (ILRV); real effective exchange rate (XUNRQ); real compensation per employee (RWCDV) and Gross national saving (USGN). The incidence of temporary employment is from the OECD Dataset: LFS—Employment by Permanency.

2 When dealing with non-stationary data in cointegration analysis, there are a number of advantages derived from the use of panels. Actually, by adding the cross section dimension, the non-stationarity of time series can be treated with greater data and power than the one provided by cross sections. The latter behaves like repeated actions of the same distribution and so, while it is known that the standard contrast Dickey-Fuller (DF) lacks of power when distinguishing the null of unit root from the stationary alternative, using the cross-sectional dimension of panel data increases the power of contrasts that are based on a simple extraction of the population under consideration.

3 The two tests differ in the formulation of the hypotheses. The trace test is one-sided (with the alternative hypothesis of having than r cointegrating vectors), on the other hand, maximum eigenvalue execute separate tests with an alternative hypothesis of r+1 cointegration vectors.

4 Properties of panel DOLS, when there are fixed effects in the cointegrating regression, have been extensively discussed by Kao and Chiang (2000).

5 Kao and Chiang (2001) demonstrated that a panel dynamic OLS estimator has the same asymptotic distribution as the type of panel FMOLS estimator derived in Pedroni (1996) and showed that the small sample size distortions for such an estimator were often smaller than certain forms of the panel FMOLS estimator.

6 Note that from our analysis we get grouped cross-section and individual coefficients. Focusing on individual countries some changes in the signs emerge, however they are not statistically significant.

7 If two series are *I*(1) and cointegrated, a causal relationship will exist in at least one direction (Engle and Granger 1987).

8 The methodology proposed by Johansen (1988, 1995) and Juselius (2006), requires estimating a Vector Error Correction (VEC) model. Unless one of the variables is weakly exogenous single equation methods cannot be employed.

9 The choice of an appropriate number of time lags is crucial for the estimation of the unrestricted VAR, which is known to be “sensitive” to the number of time lags (Banerjee et al. 1993). Ho and Sørensen (1996) find evidence in favour of using Akaike Information criterion (AIC) when a cointegration analysis is intended. Winker (1995, 2000) generalizes this model selection step to allow for different lag structures across equations. By performing the “Lag Order Selection Criteria” and considering the Akaike information and Schwarz information criterions, it suggests the use of 1 and/or 2 lags, which seems to be sense because the dataset dimension.

10 Notice that, Granger-causality is an indicator of marginal predictive ability and may not have any implications about actual economic cause and effects. That is, the finding that ER can predict TFP cannot be taken as evidence that changes in ER will produce future changes in TFP. Movements in ER might well reflect market expectations of future changes in productivity.
